# Inhibition of MDM2 homodimerization by XIAP IRES stabilizes MDM2, influencing cancer cell survival

**DOI:** 10.1186/s12943-015-0334-0

**Published:** 2015-03-26

**Authors:** Tao Liu, Hailong Zhang, Jing Xiong, Sha Yi, Lubing Gu, Muxiang Zhou

**Affiliations:** Department of Pediatrics and Aflac Cancer and Blood Disorders Center, Emory University School of Medicine, 1760 Haygood Drive, Atlanta, GA 30322 USA

**Keywords:** MDM2, XIAP IRES RNA, Cancer cell growth and apoptosis

## Abstract

**Background:**

It is known that the MDM2 protein is stabilized when it forms a heterodimer with its partner MDM4, but MDM2 protein stability in its homodimer form is not known. The MDM2 protein contains a C-terminal RING domain that not only functions as an E3 ligase to regulate ubiquitination of p53 and MDM2 itself, but also is characterized to be able to bind several specific cellular mRNAs to regulate gene expression. In this study, we evaluate whether the MDM2 protein stability is regulated by the binding of a specific small RNA (XIAP IRES mRNA).

**Methods:**

We performed chemical cross-linking and bimolecular fluorescence complementation (BiFC) assay to measure the human MDM2 protein stability in its homodimer form and the effect of XIAP IRES on MDM2 homodimerization and protein stabilization. Ubiquitination and pulse-chase assays were used to detect MDM2 self-ubiquitination and protein turn-over. Fluorescent titration and ITC were used to examine the binding between MDM2 RING protein and XIAP IRES. Western blot assay was used for determining protein expression. Clonogenic assay, WST and flow cytometry were used to test the effects of XIAP IRES, siXIAP and IR on cancer cell growth and apoptosis.

**Results:**

We found that self-association (homodimerization) of MDM2 occurs through the C-terminal RING domain of MDM2 and that the MDM2 protein becomes unstable when it is homodimerized. MDM2 homodimerization resulted in an increased function of the RING domain for MDM2 self-ubiquitination. Binding of XIAP IRES to the RING domain inhibited MDM2 homodimerization and self-ubiquitination, which resulted in stabilization of MDM2, as well as increased XIAP expression. Upregulation of XIAP and MDM2 that led to inhibition of p53 by the XIAP IRES resulted in cell growth and survival in both p53-normal and -deficient cancer cells.

**Conclusions:**

Our study identified a new IRES RNA that interacts with MDM2 protein and regulates its stabilization, which suggested that targeting of MDM2 through disruption of MDM2 protein-RNA interaction might be a useful strategy for developing novel anti-cancer therapeutics.

## Background

The MDM2 gene is an oncogene that is amplified in many human cancers [1]. Also, high levels of MDM2 expression can be observed even in malignancies without MDM2 gene amplification, as occurs in leukemia [2]. In cancer patients, MDM2 overexpression is associated with disease progression and poor treatment outcome [3-6].

The main oncogenic function of MDM2 is to inhibit the tumor suppressor p53 [7-9]; thus, p53 function becomes inactivated in MDM2-overexpressing cells, resulting in cancer cell growth and disease progression. MDM2 also plays p53-independent roles in oncogenesis. Increasing evidence suggests that even in p53-deficient cancer patients, MDM2 overexpression is still involved in cancer promotion and progression, plus resistance to treatment [10]. This is because in addition to interacting with and regulating p53, MDM2 interacts with other molecules involved in oncogenesis. For instance, MDM2 can bind to specific small RNA molecules [11-16], playing many p53-independent roles in cancer pathogenesis.

MDM2 expression is regulated in multiple ways: via gene amplification, transcriptional induction by p53, and regulation at the post-translational level by a self-ubiquitination mechanism. MDM2, a member of the RING-finger-type family of E3 ubiquitin ligases, is also a substrate of its own RING domain E3 ligase [17]. Under certain conditions, such as upon binding its partner MDMx (MDM4) and nucleic acids or p53 mRNA, the capacity of the MDM2 RING domain to target itself for ubiquitination becomes inhibited [18-20].

XIAP is an important member of the inhibitor of apoptosis protein (IAP) family. The XIAP protein binds specifically to and inhibits the activated forms of caspases 3, 7 and 9, the enzymes that induce the intrinsic (mitochondrial) apoptotic pathway, which is the major cell death mechanism that is induced by radiotherapy and many chemotherapy drugs [21]. Specific inhibition of these caspases by the XIAP protein suggests that this molecule is critical for regulating sensitivity to anti-cancer treatment. In fact, it is reported that upregulated XIAP is detected in many cancer patients and that a high level of XIAP expression is associated with resistance to chemotherapy and a poor prognosis [22-26]. The expression of the XIAP protein is uniquely regulated by an IRES-dependent mechanism at the translational level [27]. IRES-dependent translation of XIAP becomes specifically activated when cells undergo stress, such as during chemotherapy [28].

We previously found that the MDM2 RING domain protein binds to the XIAP IRES RNA, increasing IRES-mediated XIAP translation, which results in increased expression of XIAP and resistance to anticancer treatment [12]. In the present study, we evaluated whether the expression of MDM2, in particular the E3 ubiquitin ligase activity of MDM2 RING domain, is affected by its binding with the relatively small XIAP IRES RNA. We found that binding of XIAP IRES to the MDM2 RING domain protein inhibited its ability for self-association and self-ubiquitination, which increased MDM2 protein stabilization and cancer cell survival.

## Results

### MDM2 is unstable in homodimer form

Previous studies demonstrate that MDM2 is able to form a heterodimer with MDM4, and that the MDM2 protein is stabilized in the heterodimer [18]. How the protein stability of MDM2 is regulated in its homodimer form is not known. Chemical cross-linking with reagents such as DSS is widely and typically used for protein homodimerization [29]. We used DSS to treat either the neuroblastoma cell line NB-1691 and LA1-55N, having endogenous MDM2 expression, or SK-N-SH having a transfected MDM2 to study MDM2 homodimerization and to evaluate whether homodimerization affects the level of MDM2 protein expression.

**Figure 1:**
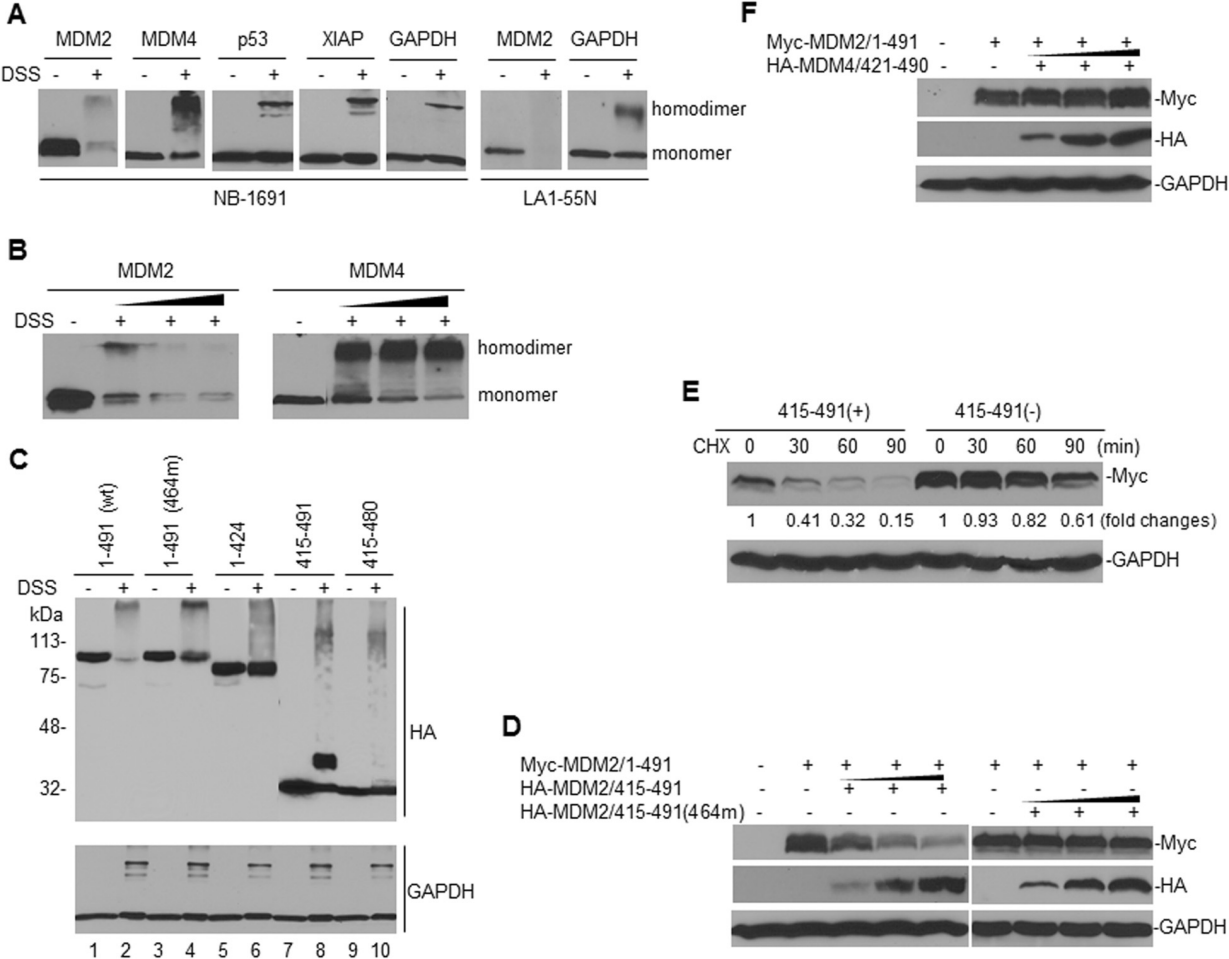
**Effect of homodimerization on MDM2 protein stability. A**, homodimerization by DSS (0.5 mM) cross-linking for 30 min, with subsequent expression of proteins as indicated, in their monomer and homodimer forms in NB-1691 and LA1-55N cells were detected by Western blot assays. **B**, dose–response expression of MDM2 and MDM4 proteins after treatment with increasing concentrations (0.25, 0.5 and 1 mM) of DSS. **C**, homodimerization by DSS and expression of transfected HA-tagged full-length MDM2 and various mutated or deleted MDM2 fragments in SK-N-SH cells, as detected by Western blot using anti-HA (tagged) antibody. **D**, SK-N-SH cells were co-transfected with consistent amounts (3 μg) of Myc-tagged full-length MDM2 (Myc-MDM2/1–491) and increasing amounts (1, 2 and 3 μg) of either HA-tagged wt-RING domain of MDM2 (HA-MDM2/415–491) or 464 mutated (464 m) RING domain of MDM2. The expression of transfected full-length MDM2 and the RING domains of MDM2 was detected by Western blot, using anti-Myc and anti-HA antibodies, respectively. **E**, turnover of transfected MDM2 (as detected by anti-Myc antibody) in SK-N-SH cells, in the presence or absence of HA-MDM2/415–491, as detected by pulse-chase assay. Numerical labels under each MDM2 band represent their relative expression levels after normalization for GAPDH, as compared with untreated (0) samples that were defined as 1 unit. **F**, a similar co-transfection and Western blot assay as described in (D) for the Myc-tagged full-length MDM2 and the HA-tagged RING domain of MDM4 (421–490).

Western blot results in both NB-1691 (wt-p53) and LA1-55N (p53-null) cells treated with DSS showed that the expression of MDM2 protein in both the homodimer and monomer forms was significantly reduced or even non-detectable in LA1-55N (Figure 1A). In contrast, the expression levels of MDM4, p53, XIAP and GAPDH in the same cells that were similarly treated with DSS did not become reduced. The data in Figure 1B represent the dose–response expression levels for MDM2 and MDM4 after treatment with DSS; which showed a remarkable downregulation of MDM2 but not MDM4 in their dimer forms, by DSS cross-linking. These results suggested that the MDM2 protein is unique in that it becomes unstable when it forms a homodimer.

DSS cross-linking in SK-N-SH cells transfected with MDM2 further confirmed that homodimerization causes MDM2 protein instability. We transfected wt-MDM2 and various mutated or deleted MDM2 fragments in SK-N-SH cells and then treated with DSS. Results indicated that DSS-induced homodimerization of the full-length (1–491) MDM2 and the RING domain (415–491), but not MDM2 molecules with a RING domain deletion (1–424), nor the RING domain having a deletion of the last 11 amino acids (415–480) (Figure 1C). This suggested that the last 11 amino acids are a key region for MDM2 self-association. Moreover, we found that homodimerization resulted in reduced protein levels of wt full-length MDM2 (Figure 1C, lane 2), while a full-length MDM2 with a mutation at 464 was reduced less (Figure 1C, lane 4). Cells with only a homodimerized MDM2 RING domain showed no decreased protein level (Figure 1C, lane 8), suggesting that MDM2 only degrades if homodimerized and if the full-length protein is available as the RING domain ligase substrate. There was less reduction of MDM2 when the 464 mutation, which provides loss of ubiquitin activity [30], was present in the homodimer. This suggested that MDM2 degradation in the homodimeric condition is likely due to self-ubiquitination. In addition, when we performed a co-transfection of the full-length MDM2 (tagged by Myc) and the RING domain of MDM2 (tagged by HA), we found that the increase in the RING domain of MDM2 decreased the transfected full-length MDM2 protein expression (Figure 1D) and stability (Figure 1E). In contrast, co-transfection of the 464-mutated MDM2 RING protein or the MDM4 RING domain did not decrease or even enhanced (co-transfection of MDM4) the expression of the transfected full-length MDM2 (Figure 1D and F). These results further suggested that homodimerization of the MDM2 RING domain protein resulted in instability of the full-length MDM2, while heterodimerization forming the MDM2/MDM4 RING complex stabilized the full-length MDM2.

### Binding of the XIAP IRES to the MDM2 RING protein inhibits MDM2 homodimerization and self-ubiquitination

**Figure 2:**
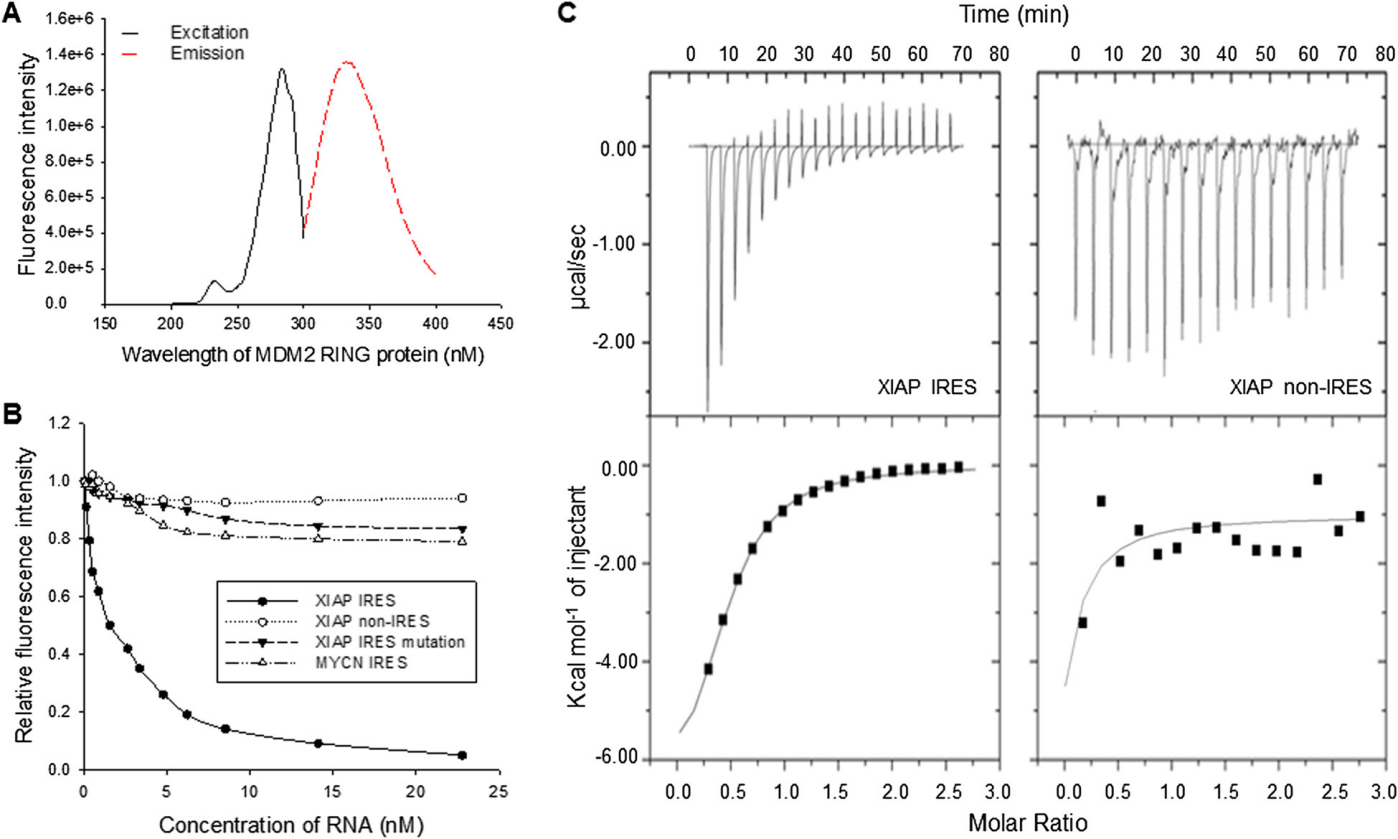
**Determination of XIAP IRES RNA binding to MDM2 C-terminal RING domain protein. A**, excitation and emission spectra of the MDM2 RING domain protein. **B**, fluorescence intensity (FI) calculation of MDM2 RING domain protein in the presence of increasing concentrations of XIAP IRES RNA and control RNAs (XIAP non-IRES, XIAP IRES mutation and MYCN IRES), relative to the FI in the absence of RNA, to derive the K_d_. **C**, thermodynamic measurement of the binding of XIAP IRES RNA (left) and XIAP non-IRES (right) to MDM2 RING protein using isothermal titration calorimetry (ITC). The upper box is the raw heating power over time and the lower box is a fit of the integrated energy values, normalized for each injection. We performed each ITC experiment three times or more.

Previous studies show that binding of nucleic acids, such as polyG, and p53 mRNA to the RING domain of MDM2 interfere with the ability of MDM2 to form homomeric complexes and the protein stabilization [19,20]. We investigated whether XIAP IRES binds to the MDM2 RING domain and inhibits MDM2 homodimerization and protein stability. We previously demonstrated the binding of XIAP IRES RNA to the MDM2 RING domain by using ^32^P-labelled probes and a RNA-binding assay [12]. In the present study, we performed fluorescent titration and ITC analyses that verified that XIAP IRES RNA indeed binds to the RING domain of MDM2 with high affinity. The GST-MDM2 RING (415–491) fusion protein has natural fluorescence, with an excitation of 280 nm and an emission of 335 nm (Figure 2A). We titrated the small XIAP IRES to the MDM2 RING protein: Results showed a Kd of 0.68 μM for the binding (Figure 2B). We also titrated to the MDM2 RING domain with several control RNAs including a non-IRES upstream XIAP 5′-UTR fragment [12], a mutated XIAP IRES and MYCN IRES, and no binding activities were detected between MDM2 and these RNAs. A similar Kd value of 0.7 μM for the binding was detected by ITC experiments (Figure 2C). These results suggest that the binding between XIAP IRES and MDM2 RING protein is specific.

**Figure 3:**
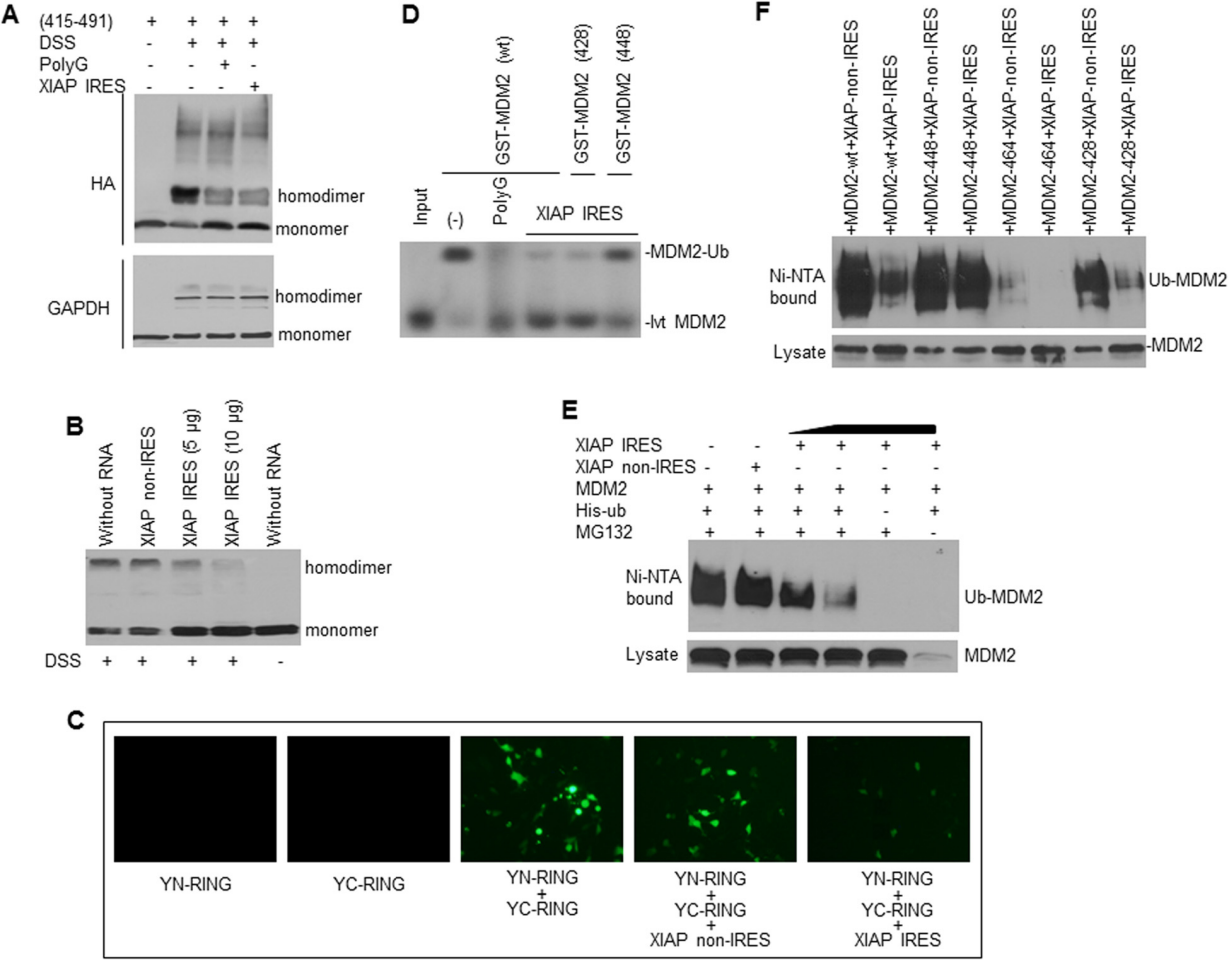
**XIAP IRES RNA inhibited MDM2 protein homodimerization and self-ubiquitination. A,** the effects of XIAP IRES RNA and PolyG (control) on the homodimerization of the MDM2 RING domain (415–491) in a co-transfection of SK-N-SH cells, as examined by DSS cross-linking. GAPDH served as a control protein. **B**, XIAP IRES decreased homodimerization of endogenous MDM2 in NB-1691 cells, as detected by DSS treatment and Western blot. **C**, Representative photographs of YFP fluorescence signal in SK-N-SH cells co-transfected with YFP N/C fragments fused to the MDM2 RING domain (YN-RING/YC-RING) in the absence and presence of XIAP IRES mRNA and XIAP non-IRES (control RNA). **D,** XIAP IRES decreased MDM2 self-ubiquitination, as examined by *in vitro* ubiquitination assays. The *in vitro*-translated (Ivt), ^35^S-labeled MDM2 was incubated under standard ubiquitination conditions in the absence (−) and presence of either XIAP IRES or polyG (control). We added the bacterially-expressed wild-type (wt) GST-MDM2 or GST-MDM2 mutated (428 or 448) fusion protein to the reactions and analyzed the reaction mixtures by SDS-PAGE, followed by autoradiography. **E**, the *in vivo* ubiquitination assay for the effect of XIAP IRES RNA on MDM2 self-ubiquitination in SK-N-SH cells co-transfected with 5 μg of each plasmid as indicated. Ni-NTA-purified, ubiquitinated MDM2 (upper) and transfected MDM2 (lower) from direct cell lysates were analyzed by Western blot. Additional controls were transfections either without His-ub or without MG132 treatment. **F**, a similar *in vivo* ubiquitination assay as in **(E)** to confirm the effect of XIAP IRES RNA on MDM2 self-ubiquitination in SK-N-SH cells transfected with different mutated MDM2 plasmids as indicated.

Now, we investigated the effect of XIAP IRES binding to the MDM2 RING protein on the homodimerization of MDM2. We performed DSS cross-linking in SK-N-SH cells transfected with the MDM2 RING domain, in the presence or absence of XIAP IRES. Our results showed that XIAP IRES, as well as polyG, inhibited the homodimerization of the transfected MDM2 RING protein (Figure 3A). Similar experiments in NB-1691 cells showed that XIAP IRES also inhibited homodimerization of the endogenous full-length MDM2, resulting in a reversal of the decrease in MDM2 levels following DSS treatment (Figure 3B).

In addition, we performed an *in vivo* bimolecular fluorescence complementation (BiFC) assay, where the MDM2 RING domain (415–491) was fused to the N (1 to 154) and C (155 to 238) terminal halves of YFP. The RING domain-mediated dimerization of two YFP fragments should reconstitute a fluorescent protein, when co-expressed in cells. As expected and shown in Figure 3C, the YN-RING or YC-RING transfections alone did not generate a signal, whereas co-transfection of the YN-RING and YC-RING produced strong fluorescence with a diffused localization in SK-N-SH cells. Meanwhile, XIAP IRES, but not the XIAP non-IRES, significantly decreased the fluorescence generated by the interaction of the YN-RING and YC-RING.

Next, we performed *in vitro* ubiquitination assays, finding that the self-ubiquitination activity of *in vitro*-translated MDM2 was inhibited by XIAP IRES, as was seen in the PolyG (positive control) reaction (Figure 3D). As also seen in Figure 3D, mutations of MDM2 448 (loss of RNA binding activity) [11] diminished the XIAP IRES-mediated inhibition of MDM2 self-ubiquitination, while the mutation of MDM2 428 (retains RNA binding activity) [12] did not, suggesting that binding to the MDM2 RING protein is essential for XIAP IRES to affect MDM2 self-ubiquitination. Furthermore, we performed co-transfection and *in vivo* ubiquitination assays and results showed that the self-ubiquitination activity of transfected MDM2 in SK-N-SH cells was inhibited by XIAP IRES in a dose-dependent manner (Figure 3E). Mutation analyses indicated that XIAP IRES failed to inhibit self-ubiquitination of MDM2 448 mutation. Mutation of 464 lost ubiquitin activity. Although mutation of 428 had reduced ubiquitin activity as compared with wt-MDM2, binding of XIAP IRES to this mutation further inhibited its activity for self-ubiquitination (Figure 3F).

### Enforced overexpression of XIAP IRES increases MDM2 expression and growth of cancer cells

**Figure 4:**
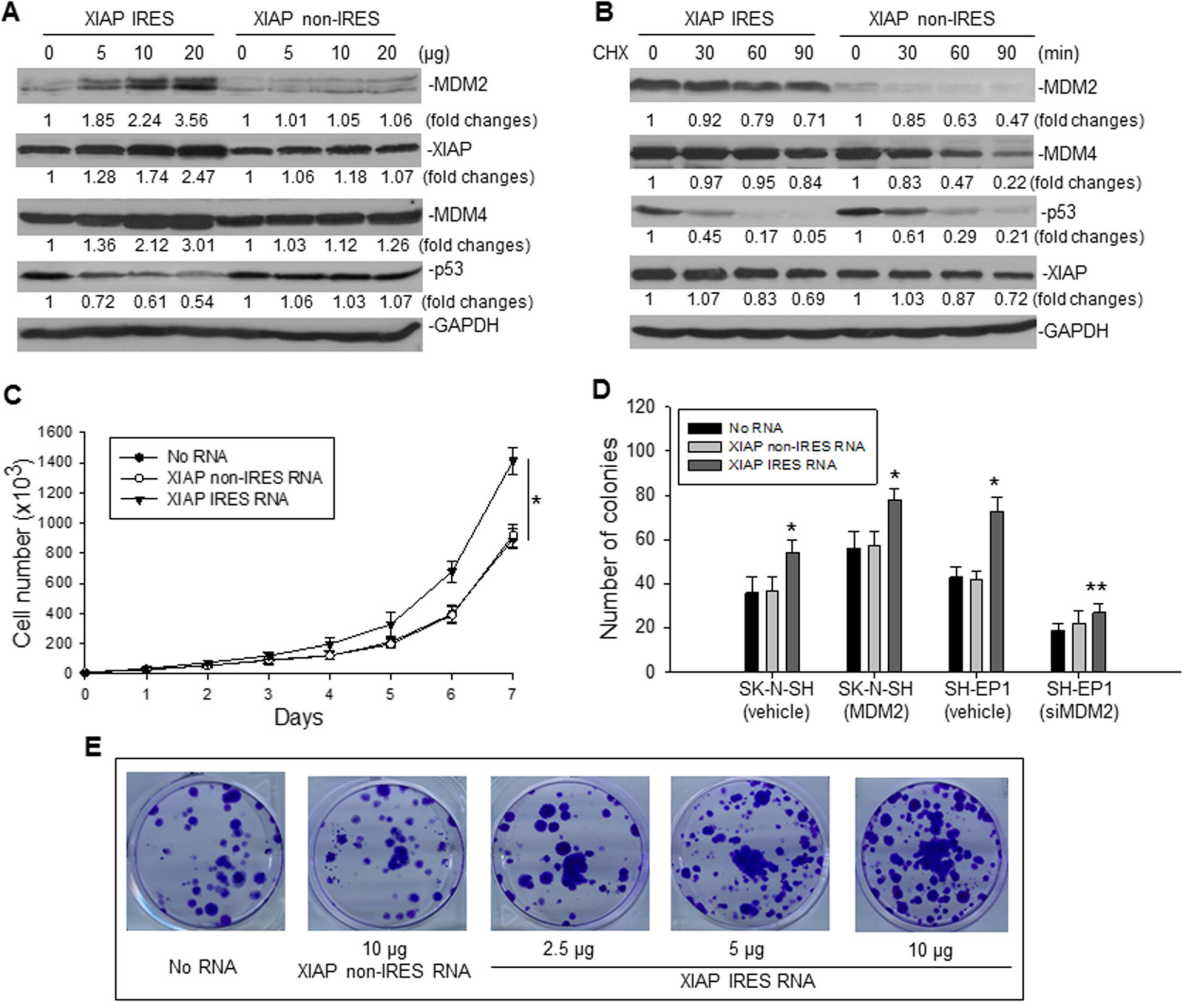
**Effect of enforced overexpresson of XIAP IRES RNA on the expression of MDM2 and XIAP and on cancer cell growth. A**, SK-N-SH cells were transfected for 24 h with the indicated amounts of pRNA-CMV3.1/Puro XIAP IRES RNA or pRNA-CMV3.1/Puro XIAP non-IRES RNA (control RNA) plasmids and the expression of MDM2, XIAP, MDM4 and p53 proteins was detected by Western blot. **B**, turnover of proteins as indicated in SK-N-SH cells transfected with XIAP IRES RNA or control RNA, as detected by pulse-chase assay. **C**, SK-N-SH cells stably transfected with either XIAP IRES RNA or control RNA expression plasmids were incubated in medium at an initial concentration of 10^4^/ml and then counted every day. Data shown: total number of cells (mean ± SD for triplicate cultures, *p < 0.05). **D**, clonogenic assay of MDM2 or vehicle-stably transfected SK-N-SH cells and siMDM2 or vehicle-stably transfected SH-EP1 cells that were co-transfected with either XIAP IRES RNA or control RNA. We seeded and cultured 200 cells with or without RNA transfection for 2 weeks, then stained the colonies and counted them under phase contrast microscopy. Data represent the mean of three independent experiments (*bars* ± SD, *p < 0.05, **p > 0.5). **E**, representative plate pictures for colony formation of SK-N-SH cells transfected with or without RNA expression plasmids as indicated.

Because binding of XIAP IRES to the MDM2 RING protein inhibited MDM2 homodimerization, which resulted in inhibition of MDM2 self-ubiquitination, we evaluated the cellular consequences of XIAP IRES-mediated inhibition of MDM2 self-ubiquitination in cancer cells. We performed a transfection of the plasmid pRNA-CMV3.1/XIAP IRES, which constitutively produced XIAP IRES RNA, to enforce overexpression of XIAP IRES in SK-N-SH cells. Transfection of XIAP IRES increased MDM2 protein expression, resulting in a concomitant decrease in p53 expression, in a dose-dependent manner (Figure 4A). Overexpression of XIAP IRES also led to a dose-dependent increase in XIAP expression, which we believe is a result of increased MDM2 expression that led to MDM2 binding to the endogenous XIAP IRES to increase its translation activity. Turnover of both MDM2 and p53 after XIAP IRES transfection was measured by pulse-chase assay. As shown in Figure 4B, transfection of XIAP IRES increased the half-life of MDM2, which was followed by enhanced degradation of p53. The turnover of XIAP protein was not changed in XIAP IRES-transfected cells as compared with control-transfected cells, suggesting that the increased XIAP expression was not due to post-translational modification.

We measured and compared the growth rate of cancer cells that were stably transfected with XIAP IRES with those transfected with XIAP non-IRES. As seen in Figure 4C, the XIAP IRES-transfected SK-N-SH cells exhibited an increased growth rate, compared to control-transfected SK-N-SH cells. We also performed clonogenic assays in SK-N-SH cells stably-transfected with MDM2 and in SH-EP1 cells stably-transfected with siMDM2, as previously established [31], in the presence or absence of XIAP IRES. XIAP IRES increased colony formation of either SK-N-SH or SH-EP1 cells expressing MDM2 but not the SH-EP1 cells with MDM2 knockdown (Figure 4D and E), suggesting that the effect of XIAP IRES on cancer cell growth is MDM2-dependent.

### Enforced overexpression of XIAP IRES increases resistance to apoptosis

**Figure 5:**
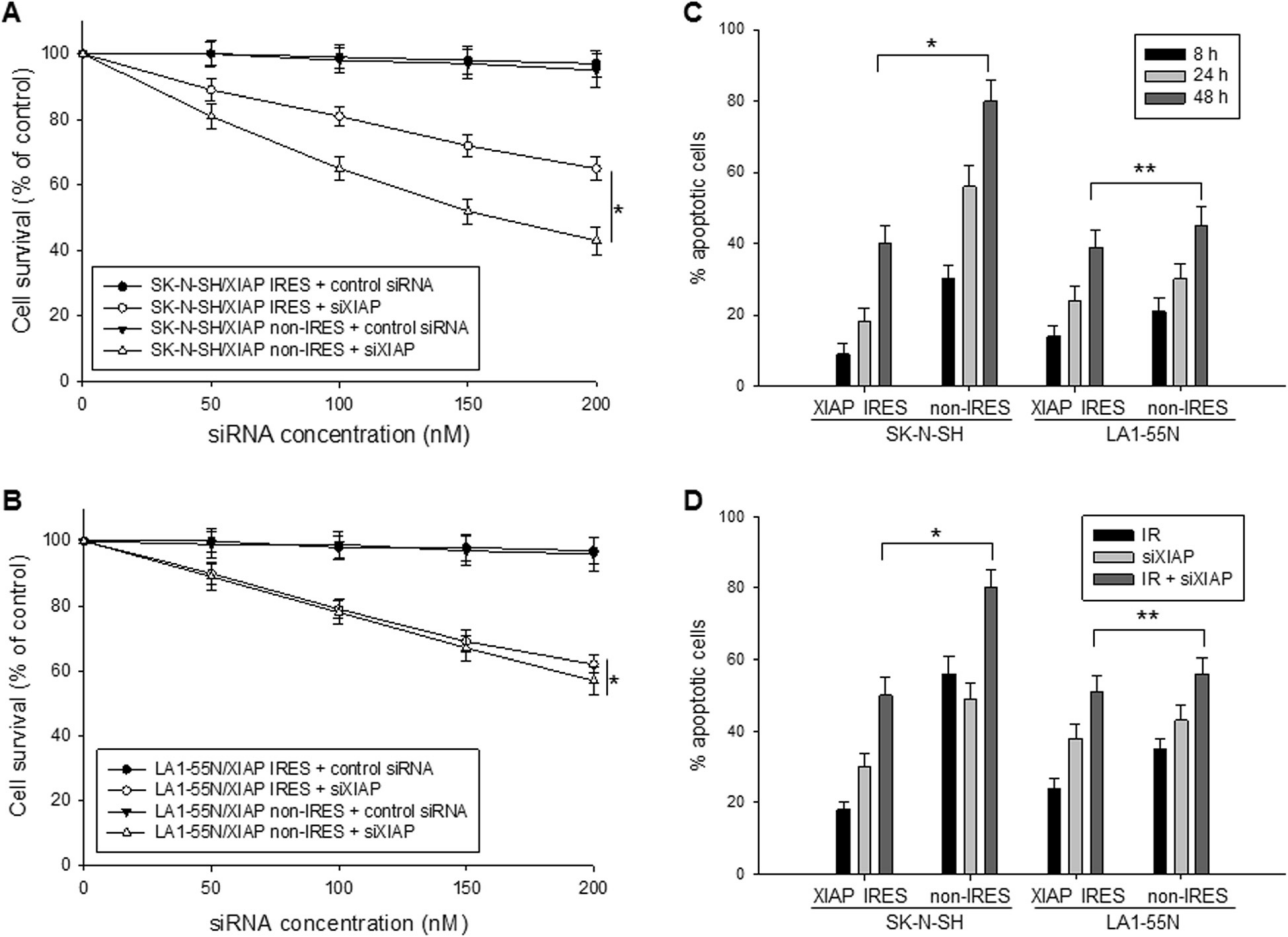
**Effect of enforced overexpresson of XIAP IRES RNA on cell apoptosis. A** and **B**, SK-N-SH (wt-p53) and LA1-55N (p53-null) cells that were stably transfected with either XIAP IRES RNA or XIAP non-IRES RNA (control) were treated with different dose of siXIAP or control siRNA for 48 h, and cell survival was determined by WST assays. Data represent the mean percentage (±SD) of cell survival from three independent experiments, *p < 0.05 **(A)**, *p* > 0.5 **(B)**. **C**, time-course of apoptosis induced by IR in SK-N-SH and LA1-55N cells that were stably transfected with either XIAP IRES RNA or XIAP non-IRES RNA. Cells were treated with 10 Gy IR for the indicated time points, and then the apoptotic cells were detected by annexin-V staining and flow cytometry. Data represent the mean percentage of annexin-V positive cells from three independent experiments, *bars* ± SD, *p < 0.01, **p < 0.05. **D**, same SK-N-SH and LA1-55N cells transfected with RNA as described above were treated with 10 Gy IR or 200 nM siXIAP alone or their combination for 24 h, and then apoptosis was detected as in (C), *p < 0.01, **p > 0.5.

We examined the effect of XIAP IRES on cancer cell apoptosis and death induced by knockdown of XIAP. Since enforced overexpression of XIAP IRES enhanced XIAP expression as well as MDM2 stabilization that led to inhibition of p53, we selected both wt-p53 and p53-null cancer cells and treated them with siXIAP, in order to know whether both MDM2-p53 and XIAP signaling pathways are involved in the effect of XIAP IRES on cancer cell survival and apoptosis. Cell lines SK-N-SH (wt-p53) and LA1-55N (p53-null) were stably transfected with XIAP IRES or XIAP non-IRES RNA (control) and then treated with siXIAP. The siXIAP inhibited cell growth or induced cell death, as detected by WST cytotoxic assay, in both SK-N-SH and LA1-55N cells that transfected either with XIAP IRES RNA or with control RNA (Figure 5A and B). However, siXIAP induced more cell death in wt-p53 SK-N-SH cells transfected with control RNA than in the same cells transfected with XIAP IRES (Figure 5A), whereas siXIAP induced almost same degree of cell death in the p53-null LA1-55N cells transfected either with XIAP IRES or with control RNA (Figure 5B). This suggested that the XIAP IRES-mediated upregulation of MDM2 and subsequently inhibition of p53 in SK-N-SH cells plays a role in protection of the cells from death induced by knockdown of XIAP.

We also performed apoptotic analysis in same SK-N-SH and LA1-55N cells transfected with XIAP IRES or control RNA and treated with ionizing radiation (IR). Similarly, IR induced much more apoptosis in wt-p53 SK-N-SH cells transfected with control RNA than in the same cells transfected with XIAP IRES. In contrast to siXIAP, IR also induced more apoptosis in p53-null LA1-55N transfected with control RNA than in the same cells transfected with XIAP IRES (Figure 5C), although the difference was smaller than that in SK-N-SH cells. Furthermore, we treated the same RNA-transfected SK-N-SH and LA1-55N cells with a combination of IR and siXIAP. A significant difference of apoptosis induction by the combination treatment between XIAP IRES and control RNA transfected cells was observed in the wt-p53 SK-N-SH cells but not in the p53-null LA1-55N cells (Figure 5D). As also seen in this figure, siXIAP seems to induce more apoptosis in XIAP IRES-transfected and less in control RNA-transfected cells, as compared to IR. Together, these results suggest that XIAP IRES binding to the MDM2 protein indeed plays a role in regulation of apoptosis.

## Discussion

MDM2 and XIAP are very important cancer-related factors: MDM2 acts as an oncoprotein, promoting cancer progression mainly through inhibition of the tumor suppressor p53; and as an anti-apoptotic protein, XIAP plays a critical role in the development of resistance to anticancer treatment via inhibition of therapy-induced caspases. Our studies demonstrated a mutual regulation of MDM2 protein and XIAP mRNA in cancer cell growth and disease progression. We previously showed that the RING domain of the MDM2 protein binds to the XIAP IRES mRNA, increasing IRES-mediated XIAP translation, which results in increased expression of XIAP and resistance to anticancer treatment [12]. In the present study, we characterized the XIAP IRES bound to the MDM2 RING domain protein, finding that the combination inhibited MDM2 homodimerization, self-ubiquitination and degradation. Enforced overexpression of the XIAP IRES RNA in cancer cells increased the expression of both MDM2 and XIAP, which led to cancer cell survival and resistance to apoptosis.

While the regulation of XIAP expression is well characterized at the translational level [27], the expression of MDM2, in particular in cancer cells, is not fully understood. Previous studies demonstrate that the expression of MDM2 is regulated at multiple levels. Overexpression of MDM2 due to genomic amplification occurs in a variety of solid human cancers [1]. In addition, overexpression of MDM2 is detected in many malignancies, such as leukemia, that lack MDM2 gene amplification [2]: The mechanism by which MDM2 is overexpressed in cancers in the absence of gene amplification is not completely known. Previous studies demonstrate that MDM2 overexpression in cancer cells is frequently associated with a wt-p53 phenotype [32,33]. Because MDM2 is a transactivational target of wt-p53, increased MDM2 expression could be a direct response to wt-p53 activity [34]. In some cancers, high levels of MDM2 expression occur due to a single nucleotide polymorphism (snp) in the MDM2 gene promoter [35]. Whether high-level MDM2 expression is also associated with the regulation of MDM2 protein stability in cancer is less understood.

MDM2 is an unstable protein that becomes ubiquitinated and degraded in an autocatalytic manner [17,36]; however, an increase in protein stability of MDM2 can be brought about by the binding of many cellular molecules to the C-terminal RING domain of the MDM2 protein. For example, the MDM2 homolog MDM4 binds to MDM2 through their respective RING domains, which increases MDM2 protein stabilization [18]. DAXX and HAUSP interact with MDM2 to form a tertiary complex, which reduces self-ubiqitination of MDM2 [37]. Nucleic acids, such as polyA or polyG, also can bind to the MDM2 RING domain and inhibit MDM2 homodimerization, as well as the capacity of MDM2 to target itself for ubiquitination [19]. The MDM2 protein stability is also regulated by a poly-ubiquitination mechanism. A recent study by Huang et al. showed that MDM2 is ubiquitinated by XIAP E3 ligase, leading to MDM2 degradation, which is involved in inhibition of autophagy, probably contributing to tumorigenesis [38].

In contrast, in the present study we found that XIAP mRNA positively regulates MDM2. We demonstrated that binding of XIAP mRNA to the RING domain of MDM2 stabilized MDM2. The expression of MDM2 protein positively regulated by mRNA binding has been previously reported by Candeias et al., who showed that binding of p53 mRNA to the RING protein of MDM2 increased MDM2 expression [20]. Our studies using transfection to enforce XIAP IRES overexpression in cells further characterized that the XIAP IRES-mediated increase in MDM2 protein expression is through the inhibition of MDM2 homodimerization, resulting in a reduction of the E3 ubiquitin ligase activity of the C-terminal RING domain that is used by MDM2 to target itself for ubiquitination and degradation. The XIAP mRNA and protein reciprocally regulate MDM2 protein stability through different mechanism, which is likely a homeostasis for MDM2 expression, suggesting that the interaction of the two factors and their deregulation may play a critical role in cancer pathogenesis.

Upon MDM2 homodimerization or its heterodimerization with MDM4, MDM2 exhibits increased E3 ubiquitin activity as compared with being in its monomer form [39]. The increased E3 activity in MDM2 homodimers is thought to function primarily in inducing MDM2 self-ubiquitination. It is proposed that in the homodimer form, one MDM2 molecule might act as a substrate, while the other one acts as the E3 enzyme to induce self-ubiquitination and degradation [40]. This notion was supported by our experimental results shown in Figure 1, in which self-association of MDM2 by cross-linking reduced the protein levels, and particularly, the expression and half-life of the transfected full-length MDM2 decreased by co-transfection of the MDM2 RING domain. In a heterodimer, the MDM2 RING domain induces ubiquitination of MDM4, but not itself [40]; although the mechanism for this selective ubiquitination is not known. Our results showed that co-transfection of the MDM4 RING domain with the full-length MDM2 did not reduce and even enhanced expression of the transfected full-length MDM2 (Figure 1E), which is consistent with a previous report that MDM2 becomes stabilized when heterodimerized with MDM4 [18].

Because the MDM2 RING domain in a heterodimer with MDM4 induces ubiquitination and degradation of MDM4, XIAP IRES RNA binding to the MDM2 RING domain might not only disrupt the formation of MDM2 homodimers, but also block the interaction between MDM2 and MDM4 that stabilizes both MDM2 and MDM4, leading to potent inhibition of p53 activity. In fact, we detected increased expression and prolonged half-life of MDM4 in enforced XIAP RNA-overexpressing cells (Figure 4A and B).

Because MDM2 is an important inhibitor of the tumor suppressor p53, and because the overexpression of MDM2 occurs in a variety of cancers, many efforts have been made to target the MDM2-p53 interaction, in order to prevent its apparent promotion of cancer and improve the power of anticancer therapies. So far, small-molecule inhibitors such as nutlin-3 and MI-219 were identified as able to efficiently block the interaction between MDM2 and p53, resulting in not only activation of p53, but apoptosis of cancer cells [41,42]; however, a limiting factor is that these small-molecule inhibitors exhibit their beneficial cytotoxic and apoptotic effects only in cancer cells bearing normal p53. Furthermore, these molecules do not inhibit MDM2, so the expression of MDM2 was even remarkably enhanced in either nutlin-3 or MI-219 -treated cells [41,42], which might still exhibit any p53-independent cancer-promoting roles, such as inducing XIAP, to diminish or even abrogate the apoptotic activity of a given small-molecule inhibitor.

In fact, a previous study has demonstrated that concomitant inhibition of MDM2 by nutlin-3 and of XIAP by small molecule antagonists synergistically induced apoptosis in wt-p53 leukemia cells [43]. Our studies including previous one [12] identify the binding between the MDM2 RING domain and XIAP IRES RNA that simultaneously increases expression of XIAP and MDM2, which provides an excellent molecular target to develop an alternative/innovative strategy for cancer therapeutics. In fact, blocking or disrupting the interaction between the MDM2 RING protein and XIAP IRES RNA by a specific antisense leads to inhibition of cancer cell growth and increased apoptosis [12]. Due to limitations in the clinical application of antisense, discovery of small-molecule compound inhibitors to block the interaction between MDM2 RING protein and XIAP IRES RNA would be very interesting. Once such inhibitors are identified, we believe that they will be able to induce much more potent apoptosis than what nutlin-3 or MI219 can: In normal p53-carrying cancer patients, treatment with a compound producing the simultaneous inhibition of MDM2 and XIAP should result not only in the activation of p53, but also in induction of caspases 3, 7 and 9. Most importantly, the same MDM2/XIAP inhibitors will almost certainly be able to induce apoptosis in p53-deficient cancer cells, if those cells express both MDM2 and XIAP.

## Methods

### Cell lines and reagents

In this study, four human neuroblastoma cell lines (NB-1691, SH-EP1, SK-N-SH and LA1-55N) were used. As previously characterized, NB-1691, SH-EP1 and SK-N-SH are wild-type (wt) p53 and LA1-55N is p53-null. NB-1691 and SH-EP1 have MDM2 overexpression, while SK-N-SH and LA1-55N have low levels of MDM2 expression [14]. These cell lines were obtained from Dr. H. Findley (Emory University) and grown in standard culture medium (RPMI 1640 containing 10% FBS, 2 mmol/L of L-glutamine, 50 units/ml penicillin and 50 μg/ml streptomycin) in incubators set at 37°C with 5% CO_2_ in air.

MDM2 antibody (SMP14) was purchased from Sigma; p53 (DO-1) was purchased from Santa Cruz; XIAP (2F1) was purchased from Abcam. MDM4 (2D10F4) antibody was purchased from LifeSpan BioSciences. The concentrations of all antibodies were used according to the manufacturers’ instruction.

### Plasmids and transfection

The HA or Myc-tagged wt and various truncated or mutated human MDM2 constructs were generated by polymerase chain reaction (PCR) and cloned them into pCMV-HA or pCMV-myc expression vectors (Clontech). A Quick Change Site-Directed Mutagenesis Kit (Stratagene) was used to mutate the MDM2 464 (substitution of Cys by Ala) and MDM2 448 (mutation of Gly to Ser), to generate plasmids that exhibited loss of E3 ligase [30] and RNA binding [11] activities, respectively. A MDM2 428 mutation (change of Ser to Gly) plasmid was generated as control. We also generated the GST-tagged, full-length MDM2 (wt and mutated) and the MDM2 C-terminal RING domain constructs by PCR and then cloned them into the bacterial pGEX expression vector. The YN-RING and YC-RING plasmids were generated by fusing the MDM2 C-terminal RING domain into the YFP/1-154 (YN) and YFP/155–238 (YC) regions, respectively, of the yellow fluorescent protein (YFP) plasmid that was provided by Dr. J. Cheng (Moffitt Cancer Center). To generate the XIAP IRES expression plasmid, we first annealed the primers 5′-GATCCTTTCACATTTTGGATTTCCTAATATAATGTTCTCTTTTTAGAAAAGGTGGA-3′ and 5′-GAAAGTGTAAAACCTAAAGGATTATATTACAAGAGAAAAATCTTTTCCACCTTCGA-3′ that contains the fragment from-34 to-62 of the XIAP 5′-UTR, which is the RNP core binding site and is bound by MDM2 [12,27], and then inserted them immediately downstream of the CMV promoter of the pRNA-CMV3.1-puro vector (Genscript, Piscataway, NJ). A control RNA plasmid was generated similarly by annealing the primers containing 28-nt of non-IRES upstream 5′-UTR of XIAP mRNA (sequence not shown) and inserting to pRNA-CMV3.1-puro vector. The pCI-His-hUbi plasmid was purchased from Addgene. Transfection of various MDM2 constructs and XIAP IRES plasmids into NB cell lines was performed in 6-well plates, using Lipofectamine^™^ 2000 reagents (Invitrogen) according to the manufacturer’s instructions. The siXIAP and control siRNA were purchased from Santa Cruz, and transfection of siRNA was carried out using the HiPerFect transfection reagent (Qiagen), following the manufacturer’s manual.

### Chemical cross-linking

Chemical cross-linking was performed using previously-described methods [44]. Briefly, cells with or without transfection of various MDM2 constructs were harvested and rinsed twice with cold PBS. The cell suspension was incubated with disuccinimidyl suberate (DSS, 20 μl of 25 mM stock solution in DMSO per 1 ml cell suspension). For the preparation of mock lysates, DMSO without DSS was added to the cells. Cross-linking was carried out for 30 min at room temperature, followed by quenching of the cross-linking with Tris-buffered saline (TBS at 25 mM, pH 7.5) for 15 min, and then cells were lysed and analyzed by Western blot.

### Immunoprecipitation and Western blot assay

Cells were lysed in a buffer composed of 50 mM Tris, pH 7.6, 150 mM NaCl, 1% Nonidet P-40, 10 mM sodium phosphate, 10 mM NaF, 1 mM sodium orthovanadate, 2 mM phenylmethylsulfonyl fluoride (PMSF), 10 μg/ml aprotinin, 10 μg/ml leupeptin and 10 μg/ml pepstatin. After centrifugation, the clarified cell lysate was separated from the pellet of cell debris and then incubated it overnight at 4°C with 15 μl Protein G plus/Protein A-agarose and 1 μg of antibodies. For Western blot, the resulting cell lysates or immunoprecipitates were resolved by sodium dodecyl sulfate polyacrylamide gel electrophoresis (SDS-PAGE), then the gel contents were transferred to a nitrocellulose filter and probed with specific antibodies, finally protein expression levels were visualized with a chemiluminescent detection system (Pierce).

### Bimolecular fluorescence complementation (BiFC) assay

The YN-RING and YC-RING fusion plasmids that contain the MDM2 RING domain, along with or without pRNA-CMV-XIAP IRES, were co-transfected into SK-N-SH cells, using Lipofectamine 2000 reagents for 24 h. The cells were cultured at 30°C for 10 h to allow maturation of the fluorophore, and then counted the MDM2 RING dimer-positive cells with visible YFP fluorescence and executed a comparison, in a blinded fashion, of the different samples.

### In vitro and in vivo ubiquitination assays

For *in vitro* ubiquitination assay, the full-length GST-MDM2 (wt and various mutations) was expressed and purified as previously described [12]. The substrate MDM2 was produced by *in vitro* translation in rabbit reticulocyte lysate, using the TNT system (Promega), in the presence of [^35^S] methionine. The *in vitro* ubiquitination assay was performed in a total reaction volume of 50 μl, consisting of 1 μl ^35^S-labeled substrate MDM2, 50 ng E1, 50 ng UbcH5, 10 μg ubiquitin and 500 ng GST-MDM2; in the absence or presence of XIAP IRES RNA that was prepared as previously described [12], in a buffer containing 25 mM Tris–HCl (pH 7.5), 100 mM NaCl, 1 mM dithiothreitol, 2 mM ATP and 4 mM MgCl_2_. After a 2-h incubation at 30°C, the mixture in SDS sample buffer was boiled, fractionated it by SDS-PAGE, and then detected by autoradiography.

For *in vivo* ubiquitination assay, cells were co-transfected with the plasmids for MDM2, His6-ubiquitin and XIAP IRES. After a 24-h incubation, the cells were treated with (or without) MG132 for an additional 6 h, and then the cells were collected from each transfection into two aliquots. One aliquot (10%) was used for conventional Western blotting to confirm the transfected MDM2 was expressed. The remaining cells (90%) were used for the purification of His6-tagged MDM2, with the aid of Ni^2+^-nitrilotriacetic acid beads, as previously described [45]. This His-tagged MDM2 was then eluted and analyzed by Western blot assay.

### Protein and RNA binding assays

Fluorescent titration and isothermal titration calorimetry (ITC) assays were carried out to confirm the binding of XIAP IRES RNA to the MDM2 RING domain protein. The GST-MDM2 RING (415–491) fusion protein has natural fluorescence, with an excitation of 280 nm and an emission of 335 nm. The XIAP IRES RNA and control RNAs including a non-IRES upstream XIAP 5′-UTR fragment, a mutated XIAP IRES and MYCN IRES (the sequences of these RNAs as described previously [12,14] were titrated to MDM2 RING domain protein. The tested RNAs were placed in the same buffer containing the MDM2 RING protein, so that the protein concentration was kept constant during titration. The steady-state fluorescence of the protein-RNA mixtures were acquired on a PTI Quanta-Master spectrometer (Photon Technology International, Birmingham, NJ), using a 3 ml cuvette. The slit widths for excitation and emission were adjusted to minimize any photobleaching of the sample, while achieving sufficient fluorescent signal intensity. The fluorescence measurements, as a function of RNA concentration, were fitted with the hyperbolic function F = F_f_ + (F_b_ − F_f_) [ligand_f_]/(K_d_ + [ligand_f_]), where F is the observed fluorescence, F_f_ is the fluorescence of unbound protein, F_b_ is the fluorescence from the protein-RNA complex, ligand_f_ is the concentration of the RNA, and K_d_ is its dissociation constant.

For the ITC assay, 20 μM of MDM2 RING protein was placed in a 400 μl buffer containing 10 mM Hepes at pH 7.2 and 150 mM NaCl, in a loading 96 DeepWell PP plate (Nunc, Thermo Fisher Scientific). A 10-fold concentration of XIAP IRES RNA (200 μM) in 120 μl of the same buffer was automatically transferred by the auto-ITC200 instrument (MicroCal, GE) into the sample cell. RNA solution (2 μl) was titrated stepwise into the protein sample cell using a syringe, for a total of 16 injections (except that the first injection was 0.4 μl). The equilibrium time between two adjacent injections was 210 s. We determined the binding stoichiometry (n), binding constant (Kd), and thermodynamic parameters (ΔH and ΔS) by fitting the titration curve to a one-site binding mode, using Origin software provided by the manufacturer.

### Pulse-chase assay

The protein turnover was assessed by a standard protein-synthesis inhibitor cycloheximide (CHX) assay. Briefly, cells were treated with 50 μg/ml CHX for different times before lysis, in the presence or absence of antisense or siRNA transfection. The concurrent expression levels of tested proteins were analyzed by Western blot as described above.

### Cell growth rate and clonogenic assays

For the growth rate test, cells were cultured in medium at an initial concentration of 10^4^/ml, using a total of 21 plates for each condition. Cells were counted each day, using a hemocytometer under a light microscope, in three (calculation of mean ± SD) of the 21 plates, so the growth rate could be determined after 7 days. A clonogenic assay to measure colony formation was used according to a previously described method [46]. Briefly, cells were harvested with treatment by trypsinization, producing a single-cell suspension, and then 200 cells were seeded into a 6-well plate and cultured for about 2 weeks. The colonies were stained with a mixture of 6.0% glutaraldehyde and 0.5% crystal violet for about 30 min, then carefully removed and rinsed with tap water. After that, the colonies were counted and calculated.

### WST assay

The cytotoxic effect of siXIAP on ALL cells was determined using the water-soluble tetrazolium salt (WST) assay. Briefly, cells cultured in 96-well microtiter plates were given different concentrations of siXIAP, for a 48-h period. Following this, WST (25 μg/well) was added and incubation continued for an additional 4 h before the optical density (OD) of the wells was read with a microplate reader (set at a test wavelength of 450 nm and a reference wavelength of 620 nm). Appropriate controls lacking cells were included, to determine background absorbance.

### Flow cytometry

For the quantitative detection of apoptotic cells, we performed annexin-V staining followed by flow cytometry. Cells with or without treatment were harvested and washed once with PBS, and then stained with FITC-annexin-V and PI, according to the manufacturer’s instructions. The cells were analyzed by flow cytometry.
